# Supplementary material for: Public perceptions of Ebola vaccines and confidence in health services to treat Ebola, malaria, and tuberculosis: Findings from a cross-sectional household survey in Uganda, 2020
Source: PLOS Glob Public Health. 2023 Dec 19;3(12):e0001884. doi: 10.1371/journal.pgph.0001884 (PMC10729951; doi:10.1371/journal.pgph.0001884)
Supplement: S1 Table — (DOCX) [file pgph.0001884.s002.docx]

**S1 Table.** Awareness of Ebola virus disease, tuberculosis, and malaria by sociodemographic characteristics, Uganda, March 2020

| **Characteristic** | **Aware of Ebola^†^**  **N=** **3,435** | | **Aware of tuberculosis^††^**  **N=3,446** | | **Aware of malaria^†††^**  **N=3,472** | |
| --- | --- | --- | --- | --- | --- | --- |
|  | N | % (95% CI) | N | % (95% CI) | N | % (95% CI) |
| **Sex** |  |  |  |  |  |  |
| Female | 2,074 | 97.9 (96.9, 98.6) | 2,083 | 96.5 (93.6, 98.0) | 2,097 | 99.7 (99.3, 99.9) |
| Male | 1,361 | 97.5 (95.5, 98.0) | 1,363 | 96.0 (94.0, 97.4) | 1,375 | 99.7 (99.0, 99.9) |
| **Head of household** |  |  |  |  |  |  |
| No | 1,689 | 97.0 (95.5, 98.0) | 1,697 | 96.2 (93.8, 97.6) | 1,709 | 99.5 (98.9, 99.8) |
| Yes | 1,746 | 98.2 (97.6, 98.7) | 1,749 | 96.5 (93.8, 98.0) | 1,763 | 99.9 (99.7, 99.9) |
| **Age (years)^¶^** |  |  |  |  |  |  |
| 15-24 | 749 | 95.4 (91.9, 97.4) | 748 | 95.7 (92.7, 97.5) | 758 | 99.4 (98.2, 99.9) |
| 25-34 | 868 | 98.8 (97.8, 99.3) | 868 | 95.9 (90.6, 98.3) | 872 | 99.7 (98.3, 99.9) |
| 35-44 | 702 | 98.1 (96.4, 99.0) | 710 | 97.5 (95.6, 98.6) | 714 | 99.8 (98.8, 99.9) |
| 45-59 | 680 | 98.2 (97.0, 98.9) | 677 | 95.0 (91.2, 97.2) | 685 | 99.8 (99.5, 99.9) |
| 60 or older | 436 | 96.3 (93.7, 97.9) | 443 | 98.3 (96.2, 99.3) | 443 | 99.9 (99.6, 99.9) |
| **Religion^ǁ‡^** |  |  |  |  |  |  |
| Christian | 3,085 | 97.6 (96.5, 98.4) | 3,096 | 96.0 (93.9, 97.4) | 3,117 | 99.7 (99.4, 99.9) |
| Others | 348 | 97.5 (94.5, 98.9) | 348 | 98.2 (94.3, 99.4) | 353 | 99.7 (97.9, 99.9) |
| **Education^ǁ^** |  |  |  |  |  |  |
| No formal education | 629 | 97.7 (95.9, 98.7) | 634 | 94.8 (91.4, 96.9) | 638 | 99.9 (99.5, 99.9) |
| Some primary | 1,721 | 97.2 (96.0, 98.0) | 1,731 | 97.0 (95.4, 98.0) | 1,742 | 99.4 (98.7, 99.7) |
| Some secondary or higher | 1,080 | 98.0 (96.6, 98.8) | 1,076 | 96.0 (92.3, 98.0) | 1,087 | 99.9 (99.8, 99.9) |
| **Residential setting^¶^** |  |  |  |  |  |  |
| Urban | 1,067 | 99.1 (98.0, 99.6) | 1,067 | 98.1 (94.5, 99.4) | 1,082 | 99.9 (99.6, 99.9) |
| Rural | 2,368 | 95.3 (93.0, 96.8) | 2,379 | 93.5 (90.8, 95.5) | 2,390 | 99.4 (98.6, 99.8) |
| **District** |  |  |  |  |  |  |
| Low Ebola risk | 1,210 | 89.4 (84.6, 92.8) | 1,214 | 96.4 (92.0, 98.4) | 1,221 | 99.6 (98.5, 99.9) |
| High Ebola risk | 2,225 | 99.2 (98.6, 99.6) | 2,232 | 96.3 (94.0, 97.7) | 2,251 | 99.7 (99.4, 99.9) |

N denotes number of respondents (un-weighted), % percentage estimates and CI confidence interval both weighted for survey sampling

† Of the total respondents in the survey (N=3,485), this analysis excluded: n=50 declined to respond.

†† Analysis excluded: n=39 declined to respond.

††† Analysis excluded: n=13 declined to respond.

§ Design-based F-statistic p values comparing prevalence across categories of a specific characteristic.

ǁ Missing values: religion (n=2), education (n=5)

¶ Age categorized and residential setting classified as per the 2016 Uganda Demographic and Health Survey provided by the Uganda Bureau of Statistics

‡ Christians include: for Anglicans, Catholics, Pentecostals, and Seventh-day Adventists
