# Supplementary material for: Public perceptions of Ebola vaccines and confidence in health services to treat Ebola, malaria, and tuberculosis: Findings from a cross-sectional household survey in Uganda, 2020
Source: PLOS Glob Public Health. 2023 Dec 19;3(12):e0001884. doi: 10.1371/journal.pgph.0001884 (PMC10729951; doi:10.1371/journal.pgph.0001884)
Supplement: S2 Table — (DOCX) [file pgph.0001884.s003.docx]

**S2 Table.** Perceptions about Ebola vaccines and confidence in health services by district-level Ebola risk profile, Uganda, March 2020

| % (95%CI) | **All districts**  **N=3,485** | **Busia**  **N=634** | **Lamwo**  **N=593** | **Arua**  **N=506** | **Greater Kampala**  **N=511** | **Kisoro**  **N=612** | **Kasese**  **N=629** | **P value** |
| --- | --- | --- | --- | --- | --- | --- | --- | --- |
| **PERCEPTIONS ABOUT EBOLA VACCINES^†^** | | | | | | | | |
| **Importance: If Uganda started having cases of Ebola, an Ebola vaccine is needed to help prevent the spread of the disease in the country**^¶^ | | | | | | | | |
| Agree | 88.2 (83.4, 91.9) | 88.3 (73.7, 95.3) | 86.0 (65.1, 95.3) | 95.1 (84.5, 98.6) | 85.3 (75.9, 91.4) | 95.2 (89.9, 97.7) | 90.4 (83.3, 94.7) | <0.001 |
| Somewhat agree | 4.0 (2.5, 6.1) | 9.7 (3.2, 26.0) | 14.0 (4.7, 34.9) | 4.9 (1.4, 15.5) | 0.7 (0.1, 4.9) | 4.1 (1.7, 9.9) | 6.1 (4.0, 9.1) |  |
| Disagree | 7.8 (4.5, 13.2) | 2.0 (0.3, 12.2) | 0 | 0 | 14.0 (7.5, 24.6) | 0.7 (0.1, 5.2) | 3.5 (1.1, 11.2) |  |
| *N* | *609* | *45* | *64* | *91* | *78* | *104* | *227* |  |
| **Acceptability: If there is an Ebola outbreak in your district, how many people in your community do you think would agree to take an Ebola vaccine if they were offered it?^¶¶¶^** | | | | | | | | |
| No one | 0.5 (0.1, 3.1) | 0 | 0 | 0 | 0.9 (0.1, 6.7) | 0 | 0.6 (0.1, 3.8) | <0.001 |
| Some people | 18.4 (15.6, 21.5) | 17.6 (7.8, 34.9) | 18.8 (9.2, 34.7) | 1.4 (0.2, 9.5) | 23.0 (19.2, 27.2) | 8.3 (4.7, 14.3) | 22.0 (14.3, 32.3) |  |
| Most people | 49.8 (44.9, 54.7) | 61.4 (45.4, 75.3) | 46.6 (28.6, 65.6) | 39.0 (26.8, 52.8) | 57.4 (49.2, 65.3) | 44.7 (36.1, 53.6) | 28.0 (23.3, 33.3) |  |
| Everyone | 31.2 (26.9, 35.9) | 21.0 (9.4, 40.6) | 34.6 (20.9, 51.3) | 59.5 (45.6, 72.1) | 18.6 (12.7, 26.5) | 47.0 (34.9, 59.4) | 49.4 (42.2, 56.6) |  |
| *N* | *611* | *45* | *68* | *89* | *77* | *103* | *229* |  |
| **Intention: If there is an Ebola outbreak in your district, how likely would you be to take an Ebola vaccine for yourself if you were offered it?^¶¶^** | | | | | | | | |
| Very likely to take it | 94.0 (90.4, 96.3) | 92.9 (82.1, 97.5) | 94.5 (82.7, 98.4) | 95.9 (90.7, 98.3) | 96.3 (85.7, 99.2) | 88.6 (78.1, 94.4) | 87.1 (82.5, 90.6) | 0.19 |
| Somewhat likely to take it | 4.0 (2.5, 6.3) | 3.2 (0.8, 11.8) | 5.5 (1.6, 17.3) | 4.1 (1.7, 9.3) | 1.5 (0.2, 10.6) | 11.4 (5.6, 21.9) | 9.4 (6.2, 14.1) |  |
| Not very likely to take it | 1.0 (0.3, 3.0) | 2.7 (0.4, 17.6) | 0 | 0 | 0.7 (0.1, 7.0) | 0 | 2.2 (0.5, 8.8) |  |
| Not at all likely to take it | 1.0 (0.3, 3.5) | 1.3 (0.2, 9.3) | 0 | 0 | 1.4 (0.2, 7.5) | 0 | 1.3 (0.2, 6.4) |  |
| *N* | *617* | *47* | *67* | *92* | *78* | *104* | *229* |  |
| **CONFIDENCE IN HEALTH SERVICES** | | | | | | | | |
| **EVD: How confident are you in the health services in your district to treat Ebola?^‡^** | | | | | | | | |
| Not at all confident | 45.3 (41.8, 48.8) | 56.8 (49.9, 63.3) | 42.6 (35.3, 50.2) | 34.2 (27.6, 41.5) | 50.4 (44.6, 56.1) | 26.7 (23.1, 30.8) | 38.5 (28.5, 49.4) | <0.001 |
| Somewhat confident | 26.1 (23.6, 28.7) | 24.6 (18.8, 31.4) | 46.1 (40.6, 51.6) | 23.9 (19.3, 29.2) | 21.2 (17.6, 25.3) | 26.8 (17.2, 39.2) | 37.5 (29.7, 49.4) |  |
| Very confident | 28.6 (25.0, 32.5) | 18.6 (14.0, 24.3) | 11.3 (6.8, 18.2) | 41.9 (33.3, 51.0) | 28.4 (22.3, 35.5) | 46.4 (34.3, 58.9) | 24.1 (18.6, 30.6) |  |
| *N* | *3,302* | *535* | *574* | *474* | *500* | *600* | *619* |  |
| **TB: How confident are you in the health services in your district to treat tuberculosis (TB/dry cough)?^‡‡^** | | | | | | | | |
| Not at all confident | 23.0 (20.5, 25.6) | 13.4 (9.1, 19.4) | 21.3 (16.4, 27.2) | 20.2 (15.0, 26.7) | 30.4 (26.1, 35.0) | 7.0 (3.8, 12.7) | 14.8 (9.9, 21.5) | <0.001 |
| Somewhat confident | 33.4 (30.4, 36.5) | 53.0 (47.9, 58.0) | 52.7 (47.3, 58.1) | 29.6 (21.3, 39.6) | 27.0 (22.2, 32.3) | 19.8 (13.4, 28.2) | 42.8 (36.5, 49.5) |  |
| Very confident | 43.6 (40.0, 47.4) | 33.6 (29.6, 37.8) | 26.0 (18.3, 35.4) | 50.1 (43.4, 56.9) | 42.6 (36.2, 49.3) | 73.1 (63.6, 80.9) | 42.4 (34.1, 51.2) |  |
| *N* | *3,330* | *602* | *581* | *453* | *496* | *609* | *589* |  |
| **Malaria: How confident are you in the health services in your district to treat malaria?^‡‡‡^** | | | | | | | | |
| Not at all confident | 7.0 (4.3, 11.3) | 7.6 (4.8, 11.9) | 4.0 (2.4, 6.8) | 7.1 (4.9, 10.3) | 7.7 (3.2, 17.6) | 1.8 (0.8, 3.9) | 7.6 (4.5, 12.6) | <0.001 |
| Somewhat confident | 27.9 (25.3, 30.7) | 48.4 (42.7, 54.1) | 58.5 (46.5, 69.6) | 19.1 (13.8, 26.0) | 27.4 (23.2, 32.0) | 7.7 (3.9, 14.7) | 19.2 (13.3, 27.0) |  |
| Very confident | 65.1 (60.7, 69.2) | 44.0 (37.4, 50.8) | 37.4 (26.8, 49.4) | 73.8 (67.2, 79.4) | 64.9 (56.9, 72.1) | 90.5 (82.4, 95.1) | 73.2 (62.6, 81.7) |  |
| *N* | *3,460* | *626* | *590* | *504* | *506* | *609* | *625* |  |

EVD: Ebola virus disease

N denotes number of respondents (un-weighted), % percentage estimates and CI confidence interval both weighted for survey sampling

§ Design-based F-statistic p values for comparing high-risk versus low-risk districts.

†Analysis on confidence on Ebola vaccine included only those who had ever heard about Ebola vaccine prior to the interview, overall=621 (Busia n=47, Lamwo n=68, Arua n=92, Kampala n=78, Kisoro n=104, Kasese n=232)

¶ Analysis excluded: 11 responded don’t know

¶¶ Analysis excluded: 3 responded don’t know, and 1 missing response

¶¶¶ Analysis excluded: 10 responded don’t know

‡ Of the total respondents in the survey (N=3,485), this analysis excluded: n=133 responded never heard of TB, n=50 declined to respond.

‡‡ Analysis excluded: those responded never heard of TB (n=116), declined to respond (n=39).

‡‡‡ Analysis excluded: those responded never heard of TB (n=12), declined to respond (n=13).
