# Supplementary material for: Public perceptions of Ebola vaccines and confidence in health services to treat Ebola, malaria, and tuberculosis: Findings from a cross-sectional household survey in Uganda, 2020
Source: PLOS Glob Public Health. 2023 Dec 19;3(12):e0001884. doi: 10.1371/journal.pgph.0001884 (PMC10729951; doi:10.1371/journal.pgph.0001884)
Supplement: S3 Table — (DOCX) [file pgph.0001884.s004.docx]

S3 Table. Sociodemographic characteristics by people who were and were not aware of Ebola vaccines, Uganda, March 2020

| Characteristics | Not aware of Ebola vaccines | | Aware of Ebola vaccines | |
| --- | --- | --- | --- | --- |
|  | N | % (95% CI) | N | % (95% CI) |
| **Sex** |  |  |  |  |
| Female | 1,679 | 83.2 (81.5, 84.9) | 338 | 16.8 (15.2, 18.5) |
| Male | 1,039 | 78.6 (76.2, 80.8) | 283 | 21.4 (19.2, 23.7) |
| **Head of household** |  |  |  |  |
| No | 1,371 | 84.0 (82.1, 85.8) | 261 | 16.0 (14.2, 17.9) |
| Yes | 1,347 | 78.9 (76.9, 80.8) | 360 | 21.1 (19.2, 23.1) |
| **Age (years)^¶^** |  |  |  |  |
| 15-24 | 611 | 85.0 (82.2, 87.5) | 108 | 15.0 (12.5, 17.8) |
| 25-34 | 686 | 80.6 (77.8, 83.2) | 165 | 19.4 (16.8, 22.2) |
| 35-44 | 553 | 80.4 (77.2, 83.2) | 135 | 19.6 (16.2, 22.8) |
| 45-59 | 516 | 78.3 (75.0, 81.4) | 143 | 21.7 (18.6, 25.0) |
| 60 or older | 352 | 83.4 (79.5, 86.8) | 70 | 16.6 (13.2, 20.5) |
| **Religion**^ǁ^ |  |  |  |  |
| Christian | 2,478 | 81.2 (79.8, 82.6) | 574 | 18.8 (17.4, 20.2) |
| Muslim | 232 | 83.2 (78.2, 87.4) | 47 | 16.8 (12.6, 21.8) |
| **Education^ǁ^** |  |  |  |  |
| No formal education | 527 | 85.1 (82.1, 87.8) | 92 | 14.9 (12.1, 17.9) |
| Some primary | 1,385 | 83.0 (81.1, 84.8) | 283 | 17.0 (15.2, 18.9) |
| Some secondary or higher | 802 | 76.6 (73.9, 79.1) | 245 | 23.4 (20.9, 26.0) |
| **Residential setting^¶^** |  |  |  |  |
| Urban | 849 | 80.5 (78.0, 82.8) | 206 | 19.5 (17.2, 22.0) |
| Rural | 1,869 | 81.8 (80.2, 83.4) | 415 | 18.2 (16.6, 19.8) |

Excludes 146 participants who did not remember if they were aware of Ebola vaccines

ǁ Missing values: religion (n=8), education level (n=5)

¶ Age categorized and residential setting classified as per the 2016 Uganda Demographic and Health Survey provided by the Uganda Bureau of Statistics
