# Supplementary material for: Public perceptions of Ebola vaccines and confidence in health services to treat Ebola, malaria, and tuberculosis: Findings from a cross-sectional household survey in Uganda, 2020
Source: PLOS Glob Public Health. 2023 Dec 19;3(12):e0001884. doi: 10.1371/journal.pgph.0001884 (PMC10729951; doi:10.1371/journal.pgph.0001884)
Supplement: Attachment — Submitted filename: scan.pdf [file pgph.0001884.s005.pdf]

PLOS Global Public Health

Public perceptions of Ebola vaccines and confidence in health services to treat Ebola, malaria, and tuberculosis: Findings from a cross-sectional household survey in Uganda, 2020

--Manuscript Draft--

Manuscript Number: PGPH-D-23-00582

Article Type: Research Article

Full Title: Public perceptions of Ebola vaccines and confidence in health services to treat Ebola, malaria, and tuberculosis: Findings from a cross-sectional household survey in Uganda, 2020

Short Title: Public perceptions of Ebola vaccines and confidence in health services to treat Ebola

Corresponding Author: Aybüke Koyuncu
Centers for Disease Control and Prevention
UNITED STATES

Schwirian
review attached

Order of Authors:
Aybüke Koyuncu
Rosalind J. Carter
Joseph Musaazi
Apophia Namageyo-Funa
Victoria M. Carter
Mohammed Lamorde
Dimitri Prybylski
Rose Apondi
Tabley Bakyaita
Amy L. Boore
Jaco Homsy
Vance R. Brown
Joanita Kigozi
Maria Sarah Nabaggala
Vivian Nakate
Emmanuel Nkurunziza
Daniel F. Stowell
Richard Walwema
Apollo Olowo
Mohamed F. Jalloh

Keywords: ebola virus disease, ebola vaccine, vaccine confidence, health systems

Abstract:

Background

Uganda used Ebola vaccines as part of its preparedness and response during the 2018-2020 10th Ebola virus disease (EVD) outbreak in neighboring Democratic Republic of the Congo (DRC). We evaluated the public's perceptions of Ebola vaccines and compared their confidence in health services to treat Ebola versus malaria and tuberculosis as part of a survey on Ebola knowledge, attitudes, and practices (KAP) conducted in March 2020.

Methods

Powered by Editorial Manager® and ProduXion Manager® from Aries Systems Corporation

Recommendation: No Recommendation Publish

Cancel | Save & Submit Later | Upload Reviewer Attachments | Proof & Print | Proceed

Reviewer Instructions

Thank you for agreeing to contribute your expert opinion to the evaluation of this manuscript.

Assessing the manuscript

As you evaluate the manuscript, consider whether it satisfies the *PLOS ONE* criteria for publication. Consult these resources for additional information:

- Reviewer Guidelines. An overview of our peer review process and what to consider in your review.
- PLOS Reviewer Center. Peer review how-to information and strategies.
- Other questions? Contact us at plosone@plos.org

Submitting your review

To complete your review, answer the questions in the review form below. (A red asterisk (*) indicates that a response is required.) Enter the main body of your review under question 5, along with any concerns about potential competing interests on the part of the authors, data availability, or research ethics. Declare your own potential competing interests under the section titled Confidential to Editor. This information is visible only to the editor and the journal office.

Review Questions

***Comments to the Author** Insert Special Character

1. Is the manuscript technically sound, and do the data support the conclusions?

The manuscript must describe a technically sound piece of scientific research with data that supports the conclusions. Experiments must have been conducted rigorously, with appropriate controls, replication, and sample sizes. The conclusions must be drawn appropriately based on the data presented.

Please select a response YES

---

*2. Has the statistical analysis been performed appropriately and rigorously?

Please select a response YES

---

*3. Have the authors made all data underlying the findings in their manuscript fully available?

The PLOS Data policy requires authors to make all data underlying the findings described in their manuscript fully available without restriction, with rare exception (please refer to the Data Availability Statement in the manuscript PDF file). The data should be provided as part of the manuscript or its supporting information, or deposited to a public repository. For example, in addition to summary statistics, the data points behind means, medians and variance measures should be available. If there are restrictions on publicly sharing data—e.g. participant privacy or use of data from a third party—those must be specified.

Please select a response YES

---

*4. Is the manuscript presented in an intelligible fashion and written in standard English?

PLOS ONE does not copyedit accepted manuscripts, so the language in submitted articles must be clear, correct, and unambiguous. Any typographical or grammatical errors should be corrected at revision, so please note any specific errors here.

Please select a response YES

*5. Review Comments to the Author

Please use the space provided to explain your answers to the questions above. You may also include additional comments for the author, including concerns about dual publication, research ethics, or publication ethics. (Please upload your review as an attachment if it exceeds 20,000 characters) (Limit 200 to 20000 Characters)

1. This is a very fine manuscript. It deals with a very important topic in a novel manner. It should serve as an example of how to compare the attitude context for a study of populations at risk. The manuscript is sound and the data well suits the conclusions.
2. The Authors used the 16th Edition of STATA. It offered the authors a lot of options for analysis which they employed well and rigorously. The data are weighted for the complex survey design, which is appropriate for this project.
3. Data availability: The data are on file at the CDC and are available on request.
4. Yes, the manuscript is quite readable and presented in standard English.

Character Count: 0

*6. PLOS authors have the option to publish the peer review history of their article (what does this mean?). If published, this will include your full peer review and any attached files.

If you choose "no", your identity will remain anonymous but your review may still be made public.

Do you want your identity to be public for this peer review? For information about this choice, including consent withdrawal, please see our Privacy Policy.

Yes

Response required
Your full name*, as it should appear in the published peer review:

*Please do not sign this review on behalf of another person. (Limit 2 to 20000 Characters)

Patricia M. Schwirian, PhD,RN,MPH

Character Count: 0

***Confidential to Editor**

1. Do you have any potential or perceived competing interests that may influence your review? Please review our Competing Interests policy and declare any potential interests that you feel the Editor should be aware of when considering your review. If you have no competing interests, please write "I have no competing interests." (Limit 20000 Characters)

I have no competing interests that could influence my review.

Character Count: 0

2. Did you receive any assistance in preparing this review (e.g. from a post-doc or graduate student)? If yes, please provide his/her name and title here. If you have chosen to reveal your identity to the authors in question 6 above, please include the name of the person who assisted you in the response to question 6 as well. (optional) (Limit 200 Characters)

I obtained consultation and input from my colleague, Dr. Kent P. Schwirian, Emeritus Professor of Sociology, The Ohio State University, who has research experience in this area.

Character Count: 0

3. If accepted, do you think this submission should be highlighted on the PLOS ONE website? PLOS ONE does not evaluate manuscripts based on perceived significance or readership. We aim to provide tools for readers to filter and evaluate our publications. (optional)

Please select a response

Yes, because of the impact of Ebola

ORCID Reviewer Credit

Note: This reviewer's default response is 'No' so this Review will not be sent to ORCID; you cannot change this as you are proxied in for this person. The Reviewer may change this after you submit the review, by going to their Completed Reviewer Assignments folder and using the 'ORCID Deposit Authorization' action link. They must log in to do so - you cannot do that by proxy.

Cancel | Save & Submit Later | Upload Reviewer Attachments | Proof & Print | Proceed
