# Supplementary material for: Public perceptions of Ebola vaccines and confidence in health services to treat Ebola, malaria, and tuberculosis: Findings from a cross-sectional household survey in Uganda, 2020
Source: PLOS Glob Public Health. 2023 Dec 19;3(12):e0001884. doi: 10.1371/journal.pgph.0001884 (PMC10729951; doi:10.1371/journal.pgph.0001884)
Supplement: Attachment — Submitted filename: Response to reviewers_EVD.docx [file pgph.0001884.s006.docx]

**Response to reviewers: “Public perceptions of Ebola vaccines and confidence in health services to treat Ebola, malaria, and tuberculosis: Findings from a cross-sectional household survey in Uganda, 2020”**

We thank the reviewers and the editor for their very useful critiques and have responded point-by-point. Our responses below are in *italics*.

Editor:

1. Please include a complete copy of PLOS’ questionnaire on inclusivity in global research in your revised manuscript. Our policy for research in this area aims to improve transparency in the reporting of research performed outside of researchers’ own country or community. The policy applies to researchers who have travelled to a different country to conduct research, research with Indigenous populations or their lands, and research on cultural artefacts. The questionnaire can also be requested at the journal’s discretion for any other submissions, even if these conditions are not met.  Please find more information on the policy and a link to download a blank copy of the questionnaire here: <https://journals.plos.org/globalpublichealth/s/best-practices-in-research-reporting>. Please upload a completed version of your questionnaire as Supporting Information when you resubmit your manuscript.

2. Please amend your detailed Financial Disclosure statement. This is published with the article. It must therefore be completed in full sentences and contain the exact wording you wish to be published.

a. State the initials, alongside each funding source, of each author to receive each grant.
b. State what role the funders took in the study. If the funders had no role in your study, please state: “The funders had no role in study design, data collection and analysis, decision to publish, or preparation of the manuscript.”
c. If any authors received a salary from any of your funders, please state which authors and which funders.

If you did not receive any funding for this study, please simply state: “The authors received no specific funding for this work.”

3. In the online submission form, you indicated that "The underlying data set cannot be made publicly available because it contains human research participant data; however, it can be made available to any interested researchers upon request. The Centers for Disease Control and Prevention is responsible for approving such requests. To request data access, one must write to the Secretary to the Committee/Head of the corresponding author (Aybüke Koyuncu) mentioning the intended use for the data, contact information, a research project title, and a description of the analysis being proposed as well as the format it is expected. The requested data should only be used for the purposes related to the original research or study". All PLOS journals now require all data underlying the findings described in their manuscript to be freely available to other researchers, either 1. In a public repository, 2. Within the manuscript itself, or 3. Uploaded as supplementary information.
This policy applies to all data except where public deposition would breach compliance with the protocol approved by your research ethics board. If your data cannot be made publicly available for ethical or legal reasons (e.g., public availability would compromise patient privacy), please explain your reasons by return email and your exemption request will be escalated to the editor for approval. Your exemption request will be handled independently and will not hold up the peer review process, but will need to be resolved should your manuscript be accepted for publication. One of the Editorial team will then be in touch if there are any issues.

*Thank you, we have completed and uploaded the PLOS questionnaire on inclusivity in global research, amended our Financial Disclosure statement, and made a de-identified version of our underlying dataset publicly available here: https://github.com/akoyuncu4/evd_uga_2020.git*

Reviewer #1:

Line 127,Define each stage of the study. You used multi-stage sampling, what the first stage consisted of, and so on.

*The methods of the survey and multi-stage sampling have been described in detail elsewhere:*

*Musaazi J, Namageyo-Funa A, Carter VM, Carter RJ, Lamorde M, Apondi R, et al. Evaluation of Community Perceptions and Prevention Practices Related to Ebola Virus as Part of Outbreak Preparedness in Uganda, 2020. Global Health: Science and Practice 2022;10.* [*https://doi.org/10.9745/GHSP-D-21-00661*](https://doi.org/10.9745/GHSP-D-21-00661)*.*

line 130, we need to define how we calculated the size of the sample. what is the prevalence of the event under study?

*We describe how the sample size is calculated in updated lines 134-138:*

*“Sample size was calculated to provide district-level estimates of the public’s knowledge, beliefs, attitudes and practices related to EVD risks. After adjusting for an expected 25% level of non-responsiveness, 640 eligible individuals from 20 clusters were approached for consent in each district.”*

*The sample size was calculated* for outcomes that are not under study in this manuscript.

line 200, I think that Poisson regression is not appropriate for this study. Poisson regression is a prediction model that applies when the target variable Y is a count variable (number of occurrences of an event during a period of time). I don't think the dependent variable here is a count variable. I think we need to use binary logistic regression. This then involves revising the methodology, results and discussion.

*Although our outcome variable is not a count variable, our use of modified Poisson models to estimate prevalence ratios for binary outcome data is based on published literature demonstrating the appropriateness of Poisson models for common binary outcomes. When the prevalence of the outcome is >10%, the odds ratio exaggerates the relative risk or the prevalence ratio.*

*Coutinho LMS, Scazufca M, Menezes PR. Methods for estimating prevalence ratios in cross-sectional studies. Revista de Saúde Pública 2008;42:992–8.* [*https://doi.org/10.1590/S0034-89102008000600003*](https://doi.org/10.1590/S0034-89102008000600003)*.*

*Barros AJ, Hirakata VN. Alternatives for logistic regression in cross-sectional studies: an empirical comparison of models that directly estimate the prevalence ratio. BMC Medical Research Methodology 2003;3:21.* [*https://doi.org/10.1186/1471-2288-3-21*](https://doi.org/10.1186/1471-2288-3-21)*.*

*Thompson ML, Myers JE, Kriebel D. Prevalence odds ratio or prevalence ratio in the analysis of cross sectional data: what is to be done? Occup Environ Med 1998;55:272–7.*

*McNutt LACWX, X. and Hafner, J, P. Estimating the Relative Risk in Cohort Studies and Clinical Trials of Common Outcomes. 2003*

Concerning table 2, I think that there are modalities that are missing from the workforce, such as disagree, somewhat agree, etc.

*The question on vaccine importance (i.e., If Uganda started having cases of Ebola, an Ebola vaccine is needed to help prevent the spread of the disease in the country) was scaled as follows in the questionnaire: Agree, Somewhat agree, Disagree, I don’t know / I’m not sure, and Declined to respond.*

*Questions pertaining to perceptions of Ebola vaccines did not always ask about agreement consistently. For example, to measure perceptions of acceptability by others in the community, participants were asked “If there is an Ebola outbreak in your district, how many people in your community do you think would agree to take an Ebola vaccine if they were offered it?”. The response choices were everyone, most people, some people, or no one.*

Reviewer #2: 

Solid introduction. The second paragraph provides great contextual information about EVD outbreak in Uganda. Please consider making this your first paragraph and moving the current first paragraph after it or later in the introduction section.

*Our introduction aims to first orient the reader on general background about management of EVD outbreaks before talking specifically about the DRC and Uganda outbreaks, therefore we have left the introduction as is.*

The authors tend to switch between ‘Ebola vaccines’ and ‘EVD vaccines’. Consider sticking with one terminology.

*Manuscript was updated to use consistent terminology, “Ebola vaccines.”*

In the third paragraph, the authors touched upon how vaccine hesitancy may be different in Uganda compared to other Western African countries. Perhaps diving more into this would add more depth to the introduction. For example, there are recent Ebola (and COVID) vaccine hesitancy papers for people in the DRC that were published within the past year.

*Thank you for this suggestion. We have added results from DRC pertaining to Ebola vaccine confidence to the introduction:*

*“Public perceptions about Ebola vaccines have been studied in various settings, and results have consistently shown high acceptability, perceived need, and future intention to accept, and acceptance of Ebola vaccines [7–12].* ***For example, 99% (95% CI: 98.5, 99.4) of healthcare workers offered Ebola vaccines in Eastern DRC self-reported accepting the vaccine during the 2018-2020 EVD outbreak, although first-offer uptake was significantly lower (70%; 95% CI: 67.1, 73.5) and many healthcare workers did not accept the vaccine until it was offered to them multiple times [12].*** *Notably, most existing evidence on the public perception of Ebola vaccines has been collected in countries involved in the West African Ebola virus epidemic* ***or 2018-2020 outbreak in eastern DRC****, and some studies have taken place during ongoing EVD outbreaks [7,10].”*

Line 61: WHO acronym is used before providing definition.

Line 65: DRC acronym is used before providing definition.

*Thank you for noting this oversight, we have defined these acronyms prior to use.*

Results. Number and proportions should be provided whenever stating results. The authors should consider being consistent in the way they report their values as sometimes only percentages are stated (lines 220-221) and other times both the number and proportion are stated (lines 231-233).

*Thank you for flagging, we have added numbers to lines 220-221. We did not add numbers for results that are weighted proportions.*

Discussion. Great work on the discussion. The manuscript did a great job focusing on Ebola KAP. The discussion could be developed more by providing more historical (in terms of success of previous ring strategies), political, and socioeconomic context and its influence on confidence in health system. Also, adding data on actual vaccination rates in the reported districts would add more substance to the discussion as well.

*We do not have data on vaccination rates since we do not have information on the denominator (i.e. the number of health facilities where Ebola vaccines were offered to healthcare workers or the total number of healthcare workers per facility). Based on available data, we know that nearly 5000 healthcare workers in Uganda were vaccinated in 165 facilities:* [*https://www.who.int/emergencies/disease-outbreak-news/item/13-june-2019-ebola-uganda-en*](https://www.who.int/emergencies/disease-outbreak-news/item/13-june-2019-ebola-uganda-en)*. We have added this to the discussion.*

References. Unable to find reference 5 and reference 20 link did not work.

*The links for these references have been updated.*

Reviewer #3: Only a small proportion of the studied population was aware of the Ebola vaccine. In addition, the authors did not compare the sociodemographic characteristics between people who were aware and not aware of Ebola vaccine. The study conclusion about the vaccine confidence drawn from the population aware of the Ebola vaccine can not stand for the whole population. Its significance is limited.

*Thank you for this suggestion, we have added a supplementary table (S3) showing sociodemographic characteristics by people who were and were not aware of Ebola vaccines.*

The analyses on the confidence in health services provided limited exciting results either.
In measures part, the authors declared "Our main outcomes were perceptions of Ebola vaccines and confidence in health services to treat 139 Ebola, malaria and tuberculosis among respondents who reported being aware of Ebola vaccines." which is inconsistent with Table 4, where all respondents were included.

*Thank you for flagging this issue. Perceptions about Ebola vaccines were only evaluated among respondents who reported being aware of Ebola vaccines, but confidence in health services were evaluated among all respondents, regardless of their awareness of Ebola vaccines. We have updated the text accordingly.*

The authors interviewed the participants using three-way classification variables and generated binary variables in the statistical analyses, which may reduce statistical performances.

*Our decision to use binary variables was due to concern about statistical performance if the sample size in each category was too small. By increasing the number of participants in each category, we hope to improve statistical performance.*

I’ve only minor comments:

Fig. 1: The figure resolution needs improvement.

*The figure resolution has been adjusted.*

Line 65: Should be 2013-2016 epidemic.

*While we acknowledge that the index case for the West African outbreak was identified in December 2013, we use the time frame of 2014-2016 for consistency with WHO publications on the West African Ebola virus disease outbreak:*

[*https://www.who.int/emergencies/situations/ebola-outbreak-2014-2016-West-Africa*](https://www.who.int/emergencies/situations/ebola-outbreak-2014-2016-West-Africa)

*No changes made.*

Line 66: Should be over 28,000 cases.

*This sentence refers to the DRC outbreak. According to the Centers for Disease Control and Prevention (citation #3) there were 3524 cases in DRC in 2018 and 130 cases in 2020 and no cases in 2019, yielding a total of 3654 cases.*

Lines 67-72: Missing reference.

*References have now been added.*

Lines 94-95: Should be 2013-2016 epidemic.

*No changes made. See comment above.*

Line 148-149: Should be 2013-2016 epidemic.

*Updated to 2014-2016. See comment above.*

Table 2: Similar to Figure 1, it would have been interesting to further investigate why confidence was diminished in health services for Ebola versus malaria and TB (e.g., lack of ETUs to support health services, low confidence in proficient staff to support health services, low confidence in the current treatments available, etc.)

*We agree this is a much-needed area of additional research for future studies. In particular, we believe qualitative research studies are critical for elucidating underlying perceptions about Ebola services. Unfortunately, we did not collect this data in our study. We have added this recommendation to our limitations section.*
